# Dosimetric impact of 3D motion-compensated SPECT reconstruction for SIRT planning

**DOI:** 10.1186/s40658-023-00525-y

**Published:** 2023-02-07

**Authors:** Laure Vergnaud, Antoine Robert, Thomas Baudier, Sandrine Parisse-Di Martino, Philippe Boissard, Simon Rit, Jean-Noël Badel, David Sarrut

**Affiliations:** 1 CREATIS; CNRS UMR 5220; INSERM U 1044; Université de Lyon; INSA-Lyon, Université Lyon 1, Lyon, France; 2 Centre de Lutte Contre Le Cancer Léon Bérard, Lyon, France

**Keywords:** Breathing motion, Dosimetry, Radioembolization, SPECT/CT, ^99m^Tc-MAA, 3D compensated reconstruction

## Abstract

**Background:**

In selective internal radiation therapy, ^99m^Tc SPECT images are used to optimize patient treatment planning, but they are affected by respiratory motion. In this study, we evaluated on patient data the dosimetric impact of motion-compensated SPECT reconstruction on several volumes of interest (VOI), on the tumor-to-normal liver (TN) ratio and on the activity to be injected.

**Methods:**

Twenty-nine patients with liver cancer or hepatic metastases treated by radioembolization were included in this study. The biodistribution of ^90^Y is assumed to be the same as that of ^99m^Tc when predictive dosimetry is implemented. A total of 31 ^99m^Tc SPECT images were acquired and reconstructed with two methods: conventional OSEM (3D) and motion-compensated OSEM (3Dcomp). Seven VOI (liver, lungs, tumors, perfused liver, hepatic reserve, healthy perfused liver and healthy liver) were delineated on the CT or obtained by thresholding SPECT images followed by Boolean operations. Absorbed doses were calculated for each reconstruction using Monte Carlo simulations. Percentages of dose difference (PDD) between 3Dcomp and 3D reconstructions were estimated as well as the relative differences for TN ratio and activities to be injected. The amplitude of movement was determined with local rigid registration of the liver between the 3Dcomp reconstructions of the extreme phases of breathing.

**Results:**

The mean amplitude of the liver was 9.5 ± 2.7 mm. Medians of PDD were closed to zero for all VOI except for lungs (6.4%) which means that the motion compensation overestimates the absorbed dose to the lungs compared to the 3D reconstruction. The smallest lesions had higher PDD than the largest ones. Between 3D and 3Dcomp reconstructions, means of differences in lung dose and TN ratio were not statistically significant, but in some cases these differences exceed 1 Gy (4/31) and 8% (2/31). The absolute differences in activity were on average 3.1% ± 5.1% and can reach 22.8%.

**Conclusion:**

The correction of respiratory motion mainly impacts the lung and tumor doses but only for some patients. The largest dose differences are observed for the smallest lesions.

## Introduction

Selective internal radiation therapy (SIRT) is a local cancer treatment used for hepatocellular carcinoma (HCC) [1], intrahepatic cholangiocarcinoma (ICC) and liver metastases (breast cancer [2, 3], neuroendocrine tumors [4], uveal melanoma [5], colorectal and pancreatic cancer [6]). It consists in injecting ^90^Y microspheres in the hepatic artery to deliver a high dose to the lesions. Before ^90^Y delivering, a planning step is performed to plan the activity to be injected [7] and to evaluate potential risk of toxicities, such as radiation pneumonitis [8, 9], following the recommendations provided in [10, 11]. During this planning step, ^99m^Tc macroaggregated albumin (MAA) is administered to the patient and the biodistribution, assumed to be the same as the microspheres [12], is assessed from SPECT/CT images. However, these images are affected by several physical phenomena or limitations such as attenuation, scatter, resolution [13, 14] and breathing movement that deteriorate the images. While the first three phenomena are routinely accounted for by reconstruction software, breathing motion is usually not corrected. Different methods have been proposed in the literature to retrieve and correct for breathing motion in SPECT images, e.g., using external breathing monitoring device [15], fluoroscopic images [16, 17] or data-driven approaches [18, 19]. Motion correction was, for example, applied to myocardial perfusion [20, 21]. However, to our knowledge, it is not used during SIRT although the liver movement due to breathing is well known [22]. Bastiaannet et al. [23] and Lu et al. [24] studied the dosimetric impact of breathing motion on numerical phantoms (physical phantoms in [25]) and showed that it is the main undesirable effect for the quantification in radioembolization. To our knowledge, this impact has only been evaluated by Santoro et al. [25] on 12 SPECT patients images with a single liver lesion, which is not representative of all patients treated by SIRT. Such an evaluation was performed on post-treatment PET imaging [26] and has shown dose differences in the lesions and in the liver [27].

In this work, we assessed on real patient images the dosimetric impact of breathing movement during SIRT treatment planning thanks to an innovative data-driven motion-compensated method that does not require any additional material during SPECT acquisition. Absorbed dose comparisons were performed between 3D and 3D motion-compensated reconstructions [19] for several regions of interest. Prescribed activities and lung shunt fractions computed with the two methods were also compared.

## Material and methods

### Patients

Data from 29 patients (14 women and 15 men) treated by radioembolization between March 2019 and July 2021 were included. Eighteen patients from this cohort were also included in the study by Robert et al. [19]. The patient numbers common to both studies are bolded in Table 1. During treatment planning, patients received 243 ± 92 MBq of ^99m^Tc-MAA and a total of 31 SPECT/CT were available. (Two patients received two treatment sessions.) Ten patients were treated for hepatocellular carcinoma (HCC), three for cholangiocarcinoma, four for metastasis of colorectal cancer (mCRC), seven for metastasis of breast cancer and five for hepatic metastasis of other cancers. Resin spheres (Sirtex™) were administered to eleven patients and glass spheres (TheraSphere™) to 18 patients. No distinction was made between patients in this study. More details are available in Table 1. The patient data included in this study comply with current regulations for human protection and GDPR regulations (MR004).

### Image acquisition and reconstruction

SPECT/CT images were acquired with a two heads GE Discovery NM CT 670, using LEHR (Low Energy High Resolution) collimator and 3/8′′ thick crystal. The acquisition consisted of 60 projections over 360°, each with 128 × 128 pixels, 4.418 mm isotropic spacing, 25 s duration for each projection, in step and shoot mode. Energy windows were centered on 140.5 keV ± 20% width for primary and 120 keV ± 5% for scatter. Images were reconstructed with two methods: conventional ordered subset expectation maximization (OSEM), denoted 3D, and motion-compensated OSEM, denoted 3Dcomp [19]. In both cases, OSEM was performed with attenuation correction, dual energy window scatter correction and depth-dependent PSF correction (resolution recovery). The 3Dcomp method first consisted in extracting a breathing motion signal from the list-mode data thanks to Laplacian eigenmaps analysis. This signal was then split into 8 respiratory phases, and the acquired projections were sorted according to the phase to which they belong. 2D affine registrations were performed between each phase and the phase selected for the reconstruction. Affine transformations were applied to the list-mode data to correct the projections. The final 3D motion-compensated image was obtained by OSEM reconstruction from all motion-corrected list-mode data. 3Dcomp reconstruction can be performed on any respiratory phases. For attenuation correction, a single 3D CT acquisition was available and it was assumed that locally the CT was acquired during one of the phases of the respiratory movement. Reconstruction was hence performed on this corresponding phase, which has been visually determined from 4D SPECT gated reconstruction [28] (except for the estimation of the breathing motion amplitude). Note that, as shown in [19], motion-compensated reconstruction leads to better image quality than 4D gated reconstruction [28] because it uses all list-mode data and does not increase noise compared to 3D. All reconstructions (3D and 3Dcomp) were performed with 15 subsets and 15 iterations per subsets. Images were reconstructed with 128^3^ voxel grid, with spacing of 4.41806 mm. All reconstructions were performed with the RTK software [29]. No spatial filters were applied in post-reconstruction.

### Volumes of interest

Seven volumes of interest (VOI) were delineated for each patient. For liver, lungs and tumor(s), manual delineations were performed on CT scans. Note that in 50% of cases, the upper quarter of the lungs is not in the field of view (15/31). The healthy liver (HL) was obtained by removing the lesions from the whole liver. The perfused liver (PL) was estimated by intersecting the volume obtained by thresholding the SPECT image with 5% of the maximum number of liver counts with the CT volume of liver. The hepatic reserve (HR) corresponded to the liver minus the perfused volume and lesions. The healthy perfused liver (HPL) was the perfused liver minus lesions [30]. When liver and lungs VOIs overlapped, voxels in common were assigned to the liver and removed from the lungs. If a patient has several distinct lesions, all are contoured.

### Breathing motion amplitude estimation

A local rigid (translation only) registration of the liver VOI was performed between inhale and exhale phases 3Dcomp reconstructions. Registrations were performed with mutual information as similarity measure and done with Elastix [31]. The breathing motion amplitude was defined as the norm of the obtained 3D translation.

### Dosimetry workflow and analysis

A dosimetry workflow based on Monte Carlo simulations was adapted from [32] to estimate the average absorbed doses for all VOI from 3D and 3Dcomp reconstructions. ^99m^Tc SPECT images were normalized according to the injected activity by considering all detected counts in the liver and lungs VOIs. Monte Carlo simulations were performed via GATE [33] with 1 MBq of ^90^Y during 1 s and then scaled according to the activity really injected. CT images were resampled to the same voxel size as the SPECT in order to reduce the simulation time. For each SPECT, a 3D dose rate map was obtained and the mean of dose rates was computed for each VOI. The final absorbed dose per VOI was estimated by considering mono-exponential decay, with ^90^Y half-life of 64 h. Statistical uncertainties of the Monte Carlo simulations were lower than 1% in all ROIs, and computation times were around 10 min [32].

To compare the estimated absorbed doses for each reconstruction, percentages of dose difference (PDD) were computed for each VOI as expressed in Eq. (1) with D_3D_ and D_3Dcomp_ the average absorbed doses estimated with the 3D and the 3D compensated reconstructions, respectively.

1 $${\text{PDD}}\left( \% \right) = \frac{{\left( {{\text{D}}_{{3{\text{Dcomp}}}} \left[ {Gy} \right] - {\text{D}}_{{3{\text{D}}}} \left[ {Gy} \right]} \right)}}{{{\text{D}}_{{3{\text{D}}}} \left[ {Gy} \right]}} \times 100$$

Doses absorbed by the lungs were estimated by Monte Carlo simulations for each patient, and lung shunt fractions (LSFs) were calculated from Eq. 2, where C_Lungs_ is the number of reconstructed counts in the lungs and C_Liver_ is the number of reconstructed counts in the liver.

2 $${\text{LSF}}\left( \% \right) = \frac{{{\text{C}}_{{{\text{Lungs}}}} }}{{{\text{C}}_{{{\text{Lungs}}}} + {\text{C}}_{{{\text{Liver}}}} }} \times 100$$

Tumor-to-normal ratios (TN ratios) were obtained from Eq. (3), where C_T_ and C_HL_ are the number of counts detected in all tumors and healthy liver, respectively. V_T_ and V_HL_ are volumes of all tumors and healthy liver, respectively. The densities of the tumors and the healthy liver are assumed to be the same, allowing us to use volumes instead of masses.

3 $${\text{TN}} = \frac{{{{{\text{C}}_{{\text{T}}} } \mathord{\left/ {\vphantom {{{\text{C}}_{{\text{T}}} } {{\text{V}}_{{\text{T}}} }}} \right. \kern-0pt} {{\text{V}}_{{\text{T}}} }}}}{{{{{\text{C}}_{{{\text{HL}}}} } \mathord{\left/ {\vphantom {{{\text{C}}_{{{\text{HL}}}} } {{\text{V}}_{{{\text{HL}}}} }}} \right. \kern-0pt} {{\text{V}}_{{{\text{HL}}}} }}}}$$

The activity to inject to the patient was computed from Monte Carlo simulations considering the absorbed dose recommendations defined in the international guidelines according the type of sphere [10, 34].

When contouring the liver and lung VOIs, some voxels may overlap. In order to verify the dosimetric impact of assigning voxels to one or the other of the two volumes, we estimated the difference in absorbed dose (DAD) between these two choices for the two available reconstructions using Eq. (4).

4 $${\text{DAD}}\left( {{\text{VOI}}} \right) = {\text{DV}}\;{\text{Liver}}\left( {{\text{VOI}}} \right) - {\text{DV}}\;{\text{Lungs}}\left( {{\text{VOI}}} \right)$$

where DV liver (VOI) is the absorbed dose estimated for the VOI when the overlapping voxels are assigned to the liver and DV lungs (VOI) when they are assigned to the lungs.

The percentage difference in activity (PDA) was computed for each patient between the 3D and 3Dcomp reconstructions as in Eq. (1) and was not affected by the choice of the value of prescribed dose. Normality tests of the distribution over all patients of absorbed dose and activity distributions were estimated for each reconstruction and assessed with a Shapiro–Wilk test before being compared either with a paired Wilcoxon’s test or with a paired t test (3D vs. 3Dcomp). These tests were also applied to compare lung dose and TN ratios with two reconstructions. In order to predict for which patient this respiratory motion correction would be really relevant, Spearman’s correlation test was applied between PDD of tumors and seven different parameters described next. The first parameter was the volume (1) of the tumor estimated from the CT. For the next two parameters, a distance map was calculated between the tumor and the borders of the liver. The minimum (2) and average (3) distance could be estimated. The other four parameters were: (4) the minimum distance between the top of the tumor and the liver in the cranio-caudal (CC) direction [25], (5) the amplitude of tumor movement in the CC direction, (6) the overall minimum distance between the center of mass of the tumor and the liver and (7) this same distance but only in the CC direction. Correlations were also tested on these parameters divided by the volume as proposed in [35].

## Results

**Fig. 1:**
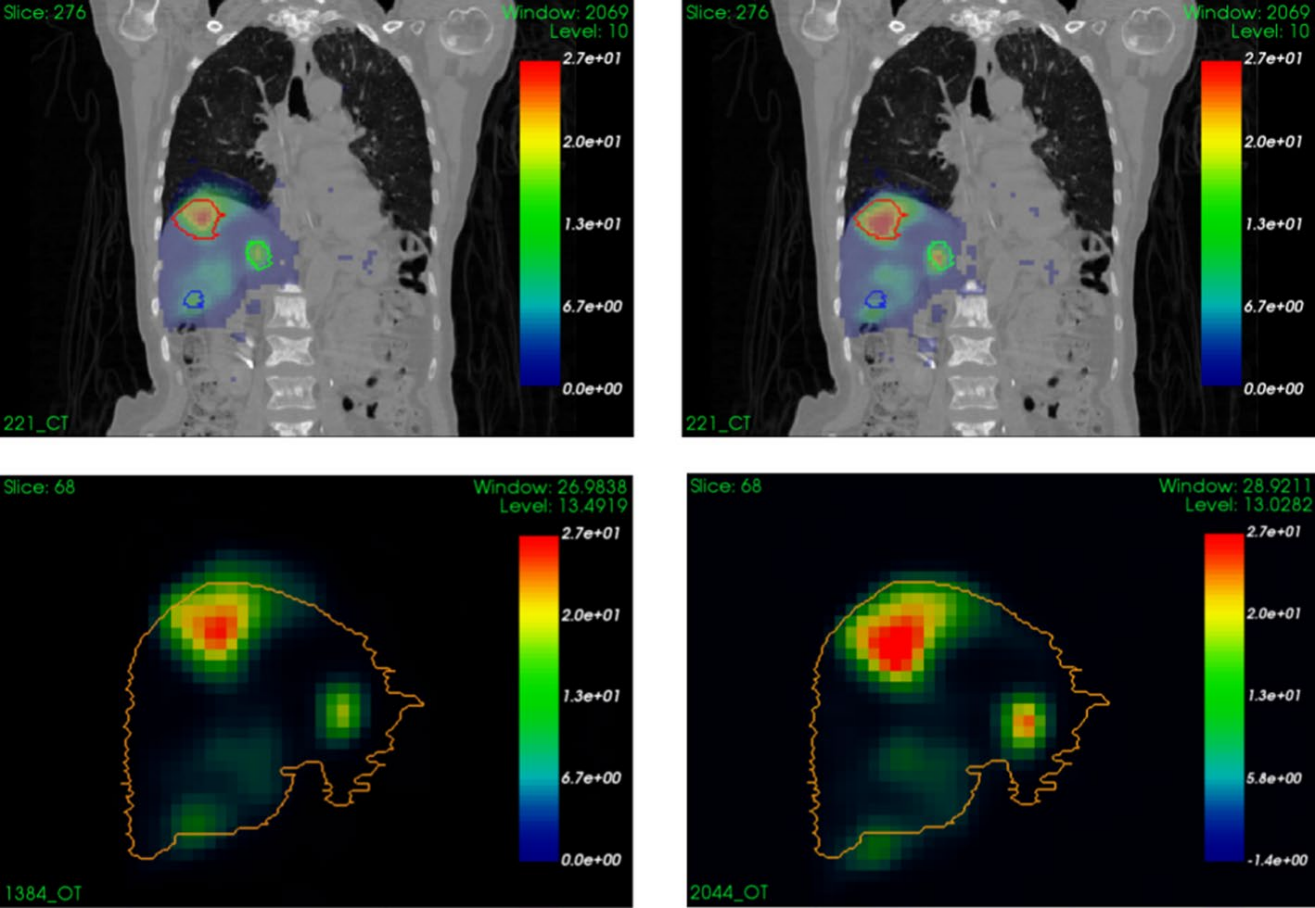
3D (left) and 3Dcomp (right) reconstructions overlapped on CT (top) and reconstructions alone. Red, green and blue contours correspond to lesion contours initially drawn on the CT image. Orange contour is the mask of the liver

The mean amplitude of the movement of the liver was 9.5 ± 2.7 mm (range 3.4–16.8 mm) and that of the tumors was 10.9 ± 2.4 mm (range 5.6–17.1 mm). If we consider only the amplitudes of liver movement associated with tumor contours, considering as many times the amplitude as there are lesions, the average amplitude of the liver was 10.8 ± 2.4 mm. No statistical significant differences were observed between the amplitude of the liver and the amplitude of the tumors (two-sided paired *t*-test *p*-value = 0.474). Figure 1 illustrates, for one patient, the 3D and 3Dcomp reconstructed images with associated contours of the tumor, showing the impact of breathing motion.

**Fig. 2:**
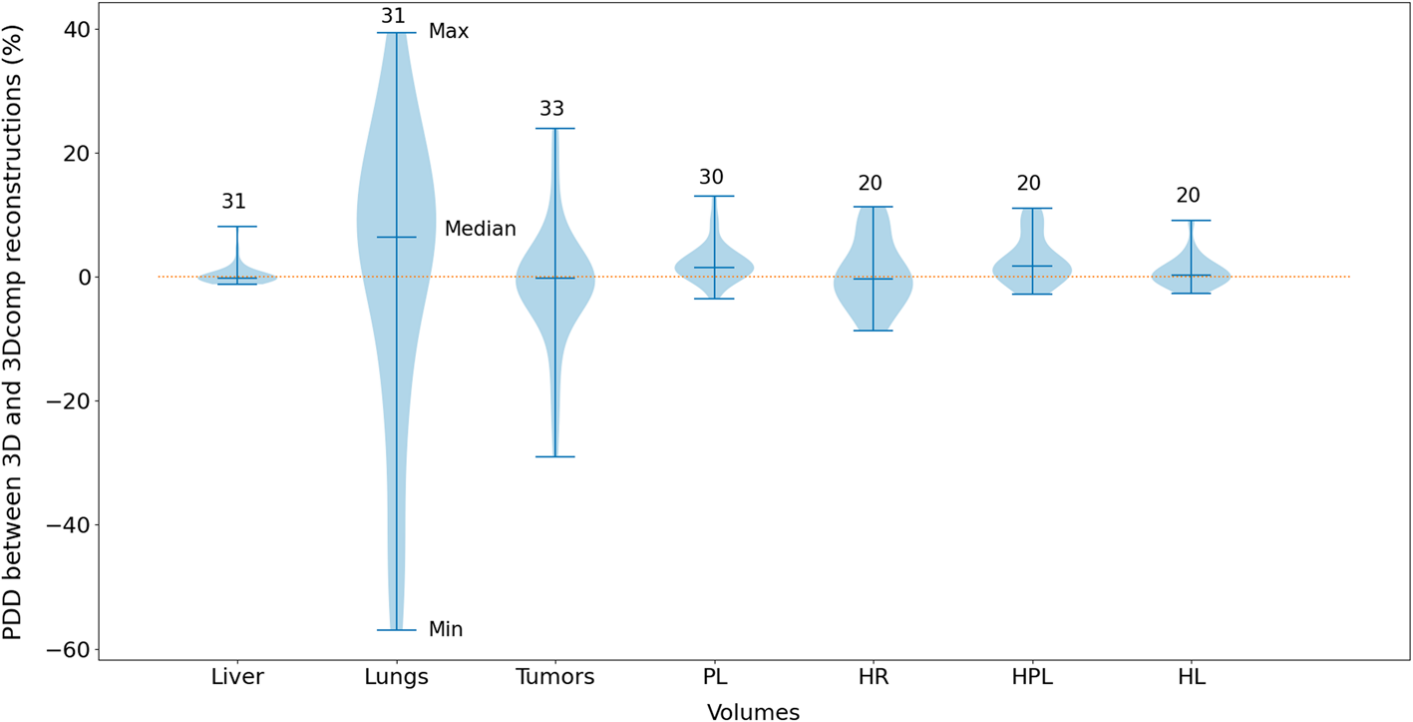
Boxplots of percentages of dose difference (PDD) between 3D and 3Dcomp reconstructions for each VOI (liver, lungs, tumors, PL, HR, HL, HPL). The number of contours used is written above each boxplot

Figure 2 shows the percentages of dose difference between absorbed doses estimated from 3D and 3Dcomp reconstructions for each VOI (liver, lungs, tumor(s), PL, HR, HPL and HL). 3D and 3Dcomp absorbed dose series of lungs, PL, HR, HPL did not follow a normal distribution (Shapiro–Wilk’s test, *p* < 0.05), while liver, HL and tumors did (only the 3D absorbed dose series). Statistically, the averages of the differences between series were not significantly different from zero (Wilcoxon’s test *p* > 0.05 for lungs, PL, HR, HPL and tumors; Student’s t-test *p* > 0.05 for liver and HL). Median, IQR, minimum and maximum values for PDD of lesions were − 0.25%, [− 3.3%, 1.1%], − 29.0% and 23.9% .

**Fig. 3:**
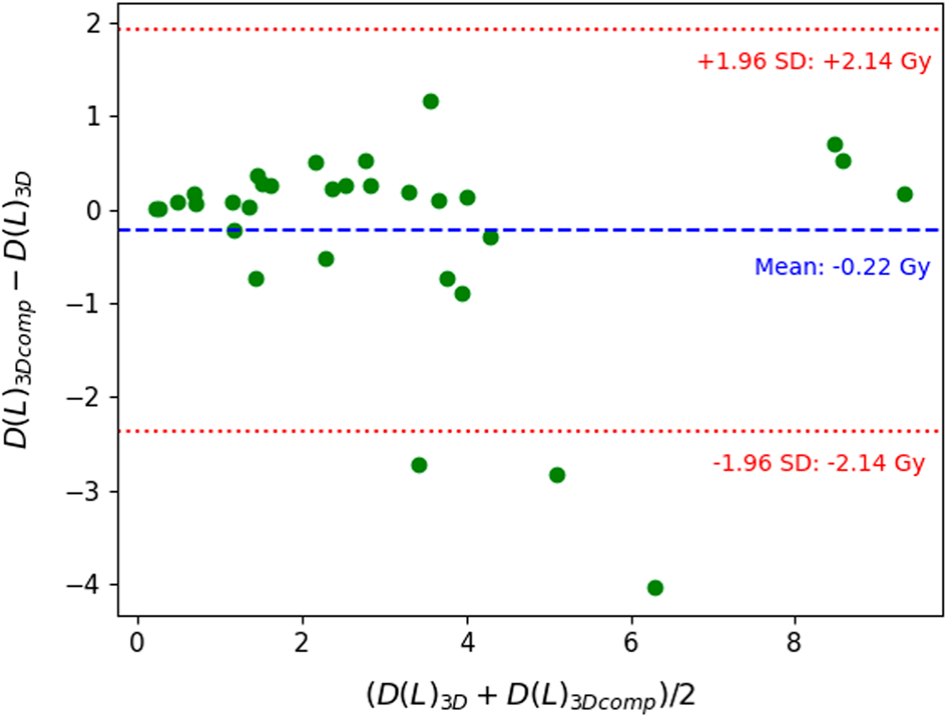
Bland–Altman plot of the lung dose (Gy) between 3D and 3Dcomp reconstructions for each patient. D(L)_3D_ and D(L)_3Dcomp_ correspond to the lung dose estimated from the 3D and 3Dcomp reconstructions, respectively

**Fig. 4:**
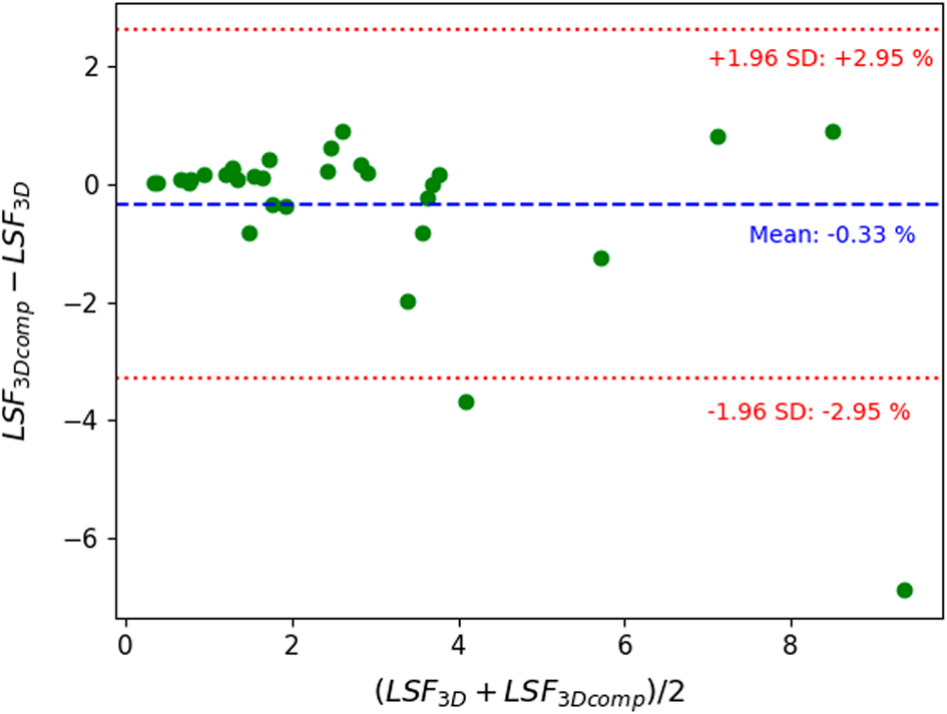
Bland–Altman plot of the LSF (%) between 3D and 3Dcomp reconstructions for each patient. LSF_3D_ and LSF_3Dcomp_ correspond to the LSF estimated from the 3D and 3Dcomp reconstructions, respectively

**Fig. 5:**
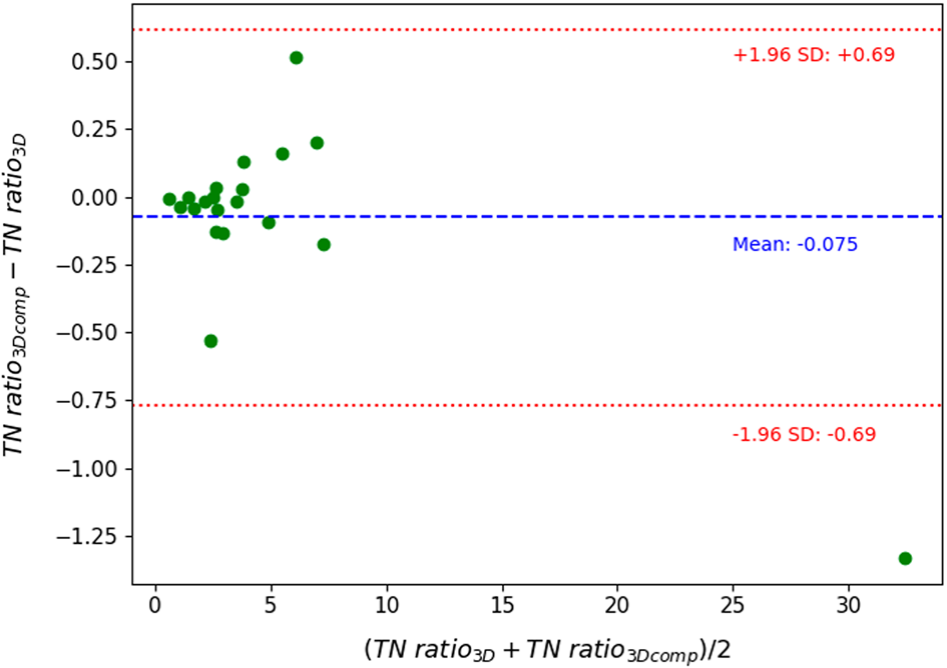
Bland–Altman plot of the tumor-to-normal liver ratio between 3D and 3Dcomp reconstructions for patients for whom lesion contours are available. TN ratio_3D_ and TN ratio_3Dcomp_ correspond to the TN ratio estimated from the 3D and 3Dcomp reconstructions, respectively

**Fig. 6:**
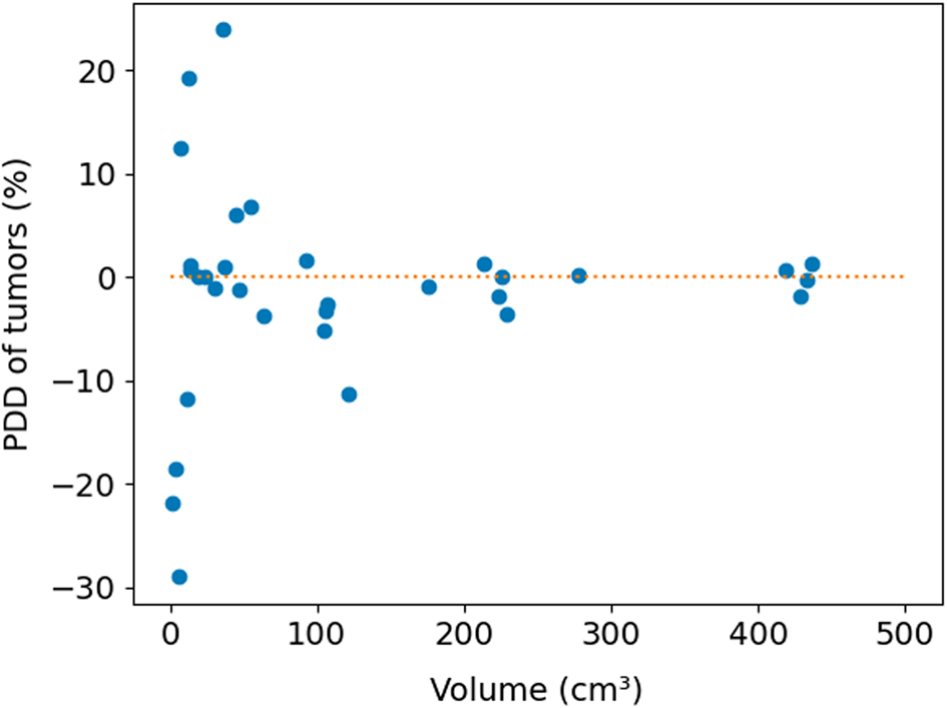
Percentages of tumor dose difference between 3D and 3Dcomp reconstructions by tumor volume

**Fig. 7:**
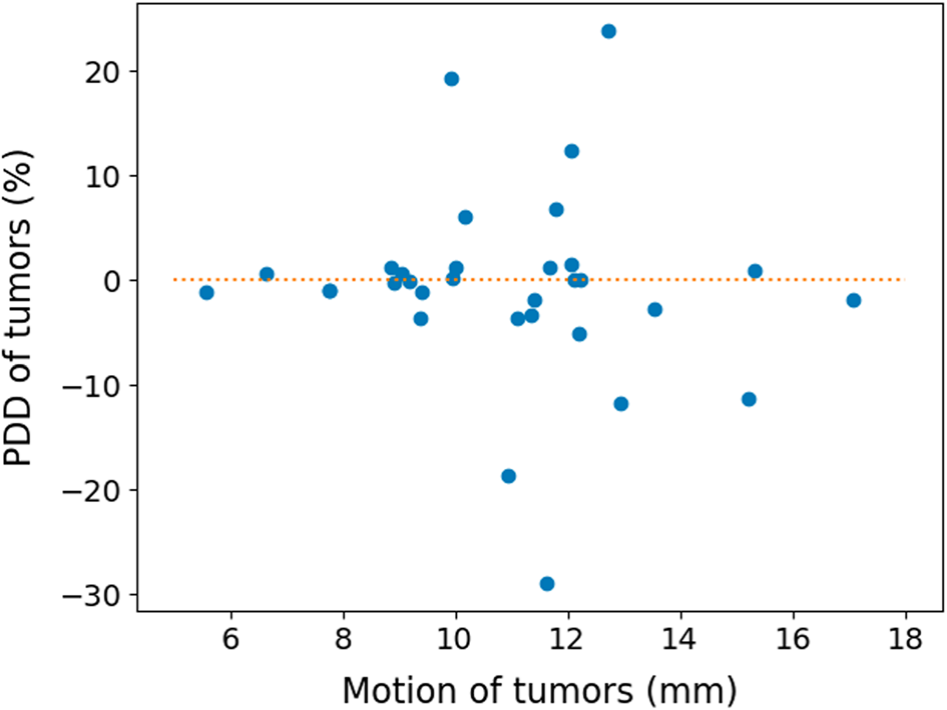
Percentages of tumor dose difference between 3D and 3Dcomp reconstructions with respect to the amplitude of the movement

**Fig. 8:**
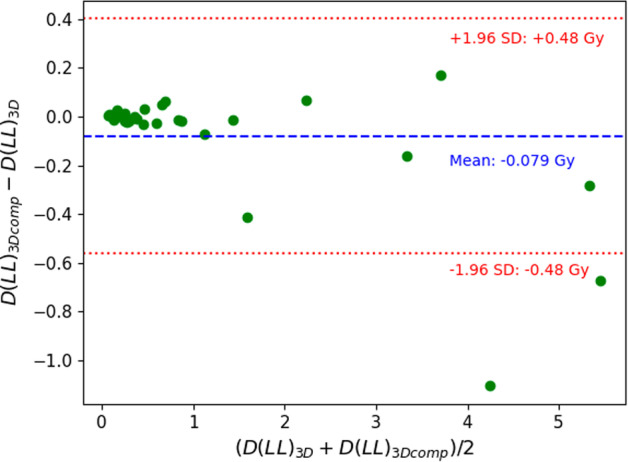
Bland–Altman plot of the left lung dose (Gy) between 3D and 3Dcomp reconstructions for each patient. D(LL)_3D_ and D(LL)_3Dcomp_ correspond to the left lung dose estimated from the 3D and 3Dcomp reconstructions, respectively

**Fig. 9:**
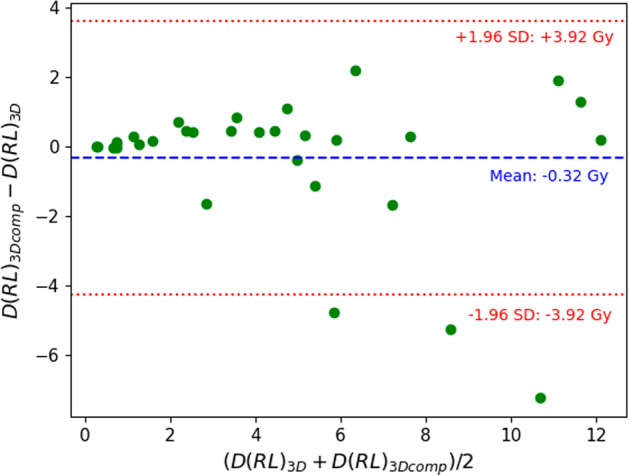
Bland–Altman plot of the right lung dose (Gy) between 3D and 3Dcomp reconstructions for each patient. D(RL)_3D_ and D(RL)_3Dcomp_ correspond to the right lung dose estimated from the 3D and 3Dcomp reconstructions, respectively

Figures 3, 4 and 5 show Bland–Altman plots between the 3D and 3Dcomp reconstructions of lung dose, LSF and TN ratio, respectively. Bland–Altman plots for the absorbed doses to the left (Fig. 8) and right (Fig. 9) lungs are available in Appendix. None of the series of differences and means follow a normal distribution: The limits of agreement are therefore given for information only. For lungs and tumors, the limits of agreements in the Bland–Altman plots were 2 Gy and 38 Gy. The data are available in Table 4 in Appendix. The mean, minimum and maximum doses estimated from the 3D reconstruction were 1.2 Gy, 0.06 Gy and 5.8 Gy for the left lung and 4.7 Gy, 0.2 Gy and 14.3 Gy for the right lung. There was no statistically significant difference for each lung with and without correction for respiratory motion (Wilcoxon’s test: *p* value > 0.05). No statistically significant correlation was found between the left and right lungs. Figures 6 and 7 represent percentages of tumor dose difference between 3D and 3Dcomp with respect to the tumor volume and amplitude of the movement of the tumors. The estimation of tumor motion was only performed for 32/33 because one of them had a too small volume which did not allow the registration between the two reconstructions and therefore the calculation of the amplitude of motion. In Fig. 6, one of the volumes was very large (1828 cm^3^) compared to the others and made it difficult to read the figure: this point was therefore removed for better readability.

For lungs, DAD (lungs) were superior to 1 Gy for only one patient for the two types of reconstruction. This discrepancy can be explained by the location of a lesion in the hepatic dome, at the border with the lungs, whose absorbed dose was estimated at 229 Gy.

For the liver, there was less than 5% of DAD(Liver) whatever the type of the reconstruction because the dose absorbed by the liver was much higher than that of the lungs (on average 3 Gy vs 46 Gy). More details are provided in Table 2 in Appendix.

**Fig. 10:**
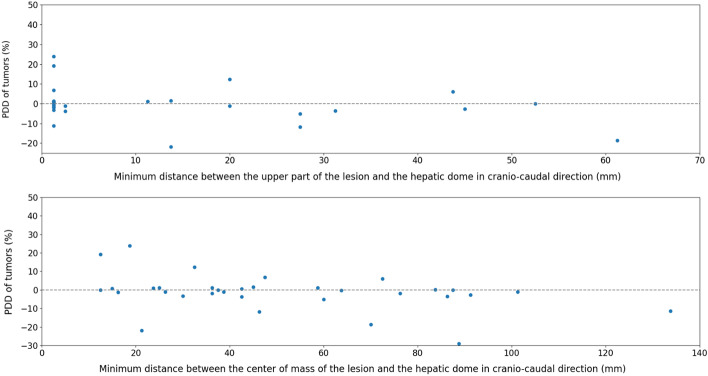
Percentages of tumor dose difference according to the minimum distance between the upper part of the lesion and the hepatic dome in cranio-caudal direction (top) and the minimum distance between the center of mass of the lesion and the hepatic dome in cranio-caudal direction (bottom)

Finally, we compared the planned injected activities with both reconstructions (Table 3). The mean and standard deviation of the absolute value of PDA were 3.1 ± 5.1% between prescribed activities obtained from 3D and 3Dcomp. The maximum absolute difference was 22.8% (1.28 GBq for 3D reconstruction vs. 1.57 GBq for 3Dcomp reconstruction) and was obtained for patient 20. Series of injected activities (3D and 3Dcomp) did not follow a normal distribution (Shapiro–Wilk’s test, *p* < 0.05). The average of the differences between prescribed activities was not significantly different from zero (*p* > 0.05 with the Wilcoxon’s test). Only two moderate statistically significant correlations (Fig. 10) could be found between PDD of tumors and 1. the minimum distance in CC direction between the upper part of the lesion and the hepatic dome (corr = − 0.38; *p* < 0.05) and 2. the minimum distance of the center of mass of the lesion in the CC direction and the hepatic dome (corr = − 0.38; *p* < 0.05).

## Discussion

This paper presents the evaluation of the dosimetric impact of respiratory movement during SIRT planning. We worked on real patient data with different characteristics (number of tumors, volumes, localizations, pathologies, type of spheres) representative of the diversity of what is found in the clinic. For each, we compared the estimated doses from the 3D and 3Dcomp reconstructions as well as the TN ratio and the activity to be injected.

Few studies have been published on the subject. In [23, 24], the authors evaluated dosimetric impacts of motion by performing simulations from digital XCAT phantoms with different sets of parameters (body type, lung shunt fraction (LSF), tumor volume and localization). Likewise, Santoro et al. [25] tested a novel method to compensate for motion on a modified CIRS dynamic phantom and applied it on the data of twelve selected patients (HCC, single lesion). However, the realism of simulations and phantoms is limited (perfect respiratory cycle and simplifications in the movement, characteristics of each patient, assumption of a perfectly compensated scatter [24]). In contrast to our method based on list-mode data, Santoro et al. [25] corrected for respiratory motion by realigning the barycenters of the lesions in the projections with each other before realigning them on the CT to improve attenuation correction and VOI definitions [36]. The latter registration consisted to have the same distance between the hepatic dome defined on the CT and the top of the lesion on two imaging modalities (SPECT and CT). However, lesion contours were obtained by thresholding on projections, themselves impacted by the respiratory movement (15 s/projection vs 5 s for one respiratory cycle): They are dependent on the distribution of the detected counts and influence the registration with the CT. Here, a 4D gated reconstruction was performed to select the phase that most visually matches the CT to avoid the impact of the breathing movement. Then, a 3Dcomp reconstruction was performed to take into account all detected counts. We assumed that CT acquisition is fast enough (10–15 s in total) to correspond locally to a respiratory phase (in the region of the hepatic dome), considering that the residual motion during the CT acquisition is low. The exact impact of this assumption is currently unknown. 3Dcomp reconstruction using other breathing phases is feasible, but they will be marred by errors because of a mismatched CT, as also observed in [36]. In both studies, VOI (liver, lungs and tumors) were delineated on CT to obtain anatomical contours independent of the distribution of the radiopharmaceutical. Although some of the patients included in the Robert et al.’s [19] study are also included in this one, the results are not comparable. Firstly, the tumors are not segmented in the same way (CT vs. thresholding) and the compensation of the respiratory movement was only carried out on the end-expiratory phase in their study, unlike ours.

The median PDD was close to zero for all VOI considered except for the lungs (6.4%). The estimated absorbed doses to the lungs are overall higher with 3Dcomp reconstruction than with 3D reconstruction. Lu et al. [36] proposed an explanation of over- and underestimation: Counts in the liver that border on the lungs can be reconstructed in the lungs and not in the liver. Similarly, counts in the lungs can be reconstructed outside the lungs. Some CT images can also have motion artifacts which can be taken into account when contouring the liver (segmented livers have a larger volume than real liver volumes): This could explain the low impact of motion on the lung doses despite the movement of the liver. This is equivalent to taking an extra margin around the liver [14, 37] to account for respiratory motion. There was no statistically significant difference for lung doses between the two reconstructions (same for LSF) which is in agreement with the results of Bastiaannet et al. [23] for the LSF. However, Lu et al. [24] showed that the LSF was overestimated when the respiratory motion was not corrected. The lesions considered are only located in segments V, VI and VIII of the liver unlike ours and in particular at the level of the hepatic dome where larger dose differences are expected. Moreover, the ranges of motion used in the simulations are higher than those estimated for our patients: between 1 and 2 cm versus 0.9 cm in the cranio-caudal direction and between 0.6 and 1.2 cm versus 0.4 cm in the antero-posterior direction that could also explain differences in conclusions. There was no significant difference in the TN ratio between the two reconstructions in contrast to other authors who found an underestimation of the TN ratio in the absence of respiratory motion correction [23, 24]. In the article by Bastiaannet et al. [23], the lesions have a volume of less than 35 mL, in contrast to those of our patients (between 1.7 and 1828 mL). The volume seems to have an impact on the error made in the absence of correction of the respiratory movement (Fig. 6): The smaller the volume, the greater the error [24, 27]. For this type of system, the central spatial resolution without scatter for ^99m^Tc at 10 cm with a LEHR collimator is 7.4 mm [38]. This implies that the partial volume effect can be particularly impactful for volumes whose dimensions are less than 2.5 times the spatial resolution, i.e., 3.3 mL. The majority of the lesions studied have a larger volume: The partial volume effect alone does not explain the dose differences observed between 3D and 3Dcomp for small volumes. The tumors had very diverse characteristics among patients, for example, in terms of volume and shape. These are likely explanations for the discrepancies observed for the TN ratio. Other studies, focused on the breathing motion impact in post-treatment [26, 27] (mathematical models and patient data), also showed that the doses to the lesions were underestimated.

On patient cohort, Santoro et al. [25] compared mean absorbed doses of lungs and tumors and showed a variability of ± 4 Gy and ± 50 Gy, respectively, which is consistent with our results (± 2 Gy and ± 38 Gy). The impact of respiratory movement on the activity to be prescribed does not appear to have been studied previously.

In this study, no significant average of differences was found between the estimated activities with each reconstruction (min: − 8.3%; median: 0.4%; max: 22.8%). Only two significant moderate correlations (*p* < 0.05) were found between the PDD of tumors and (1) the minimum distance between the upper part of tumor and the liver in the CC direction and (2) the minimal distance between the center of mass of the lesion and the liver in the CC direction. Globally, the means of the differences between 3D and 3Dcomp reconstructions for the absorbed doses and in particular the lungs, the TN ratios and the prescribed activities are not statistically significantly different from zero. However, for some patients, absolute lung dose differences between 1 and 4 Gy (4/31) were observed, which could change the management of patients in cases where the lung dose is close to the recommended dose tolerance limit. In this study, all patients had a low pulmonary shunt (maximum 3D lung dose: 9.2 Gy) compared to the recommended limits, so that management was not impacted by the correction of respiratory motion. For example, for patient 10, there is a factor of two between the estimated lung doses between the 3D and 3Dcomp reconstructions (8 Gy vs 4 Gy). Note that in this study, when liver and lung voxels overlapped, they were assigned to the liver. We assessed the dose difference if voxels were assigned to the liver or lungs and observed small dose differences compared to the doses in the VOIs (maximum absolute value for liver: 2.9 Gy and 1.2 Gy for lungs) with the exception of one patient for lungs. We chose to assign the voxels to the liver in order to avoid overestimating the absorbed dose to the lungs in cases where tumors are located in the liver dome. In addition, Robert et al. [19] showed that the recovery activity was better estimated with the 3Dcomp than with the 3D and 4D gated reconstructions. We still, therefore, consider relevant to apply a correction for respiratory movement in order to improve quantification.

The CT acquisition is a limitation of this work. Indeed, it has a preponderant role in the quantification because it influences the attenuation correction but also the accuracy of the VOI if the CT and the SPECT are not correctly aligned. Here, only a CT acquired in free breathing was available. We therefore assumed that locally (upper part of the liver), the CT was acquired in a single phase in order to perform a 3Dcomp reconstruction. The selection of the phase was done visually using the CT, and there may be uncertainty even though double validation has been done. However, a quantitative approach such as choosing the phase with the highest number of counts in the segmented lung mask on CT may be very sensitive to the segmentation and low number of counts in this region. In this case, the choice of criterion needs to be robust. Ideally, a 4D CT should be acquired because, in some cases, respiratory motion can create artifacts in the 3D CT. If this acquisition is not possible, we recommend acquiring breath-hold CT in order to perform the 3Dcomp reconstruction at the same respiratory phase.

A second limitation was the limited number of patients included. Indeed, patients’ data were heterogeneous (shape and volumes of tumors, pathologies, number of tumors) which makes it more difficult to establish with certainty the presence or absence of correlations. A larger-scale study could answer this question by grouping patients according to their characteristics. Segmentations of tumors were not available for all patients; therefore, the number of data available depends on VOI: Only 20 treatments out of 31 were considered to compute PDD of tumors, HL, HPL and HR contrary to liver, lungs and PL where all treatments were taken into account. Finally, multiple injection sites were required to treat some patients: In this case, we performed the calculations assuming that they had received a single overall injection.

A third limitation of this study was the relationship between the biodistribution of ^99m^Tc-MAA and ^90^Y microspheres. Indeed, several authors reported a good correlation of doses to tumors in the case of HCC but have shown a weaker correlation in the case of metastatic disease [39, 40]. Similarly, the positioning of the catheter between the pre-treatment and treatment stages may influence the agreement of the predicted and measured doses [41]. The differences between 3D and 3Dcomp dosimetry were not compared to the dosimetric differences obtained between ^99m^Tc and ^90^Y as this was not the focus of this study, but it is a point that would be of interest later.

## Conclusion

In this study, we demonstrated the feasibility of correcting respiratory motion on real pre-treatment SPECT images for radioembolization. The method does not need additional external devices or acquisition changes. We have shown that this correction impacts the absorbed dose to the lungs (the median percentage dose difference between 3Dcomp and 3D was 6.4% for lungs) which could impact the management of some patients in cases where the dose is close to the recommended dose tolerance limit. This correction also affects the tumor dose, where the largest dose differences were estimated for the smallest lesions.

## Data Availability

Data and materials are available from the corresponding author on reasonable request.

## Appendix

(See Figs. 8, 9, 10 and Tables 1, 2, 3, 4).

**Table 1:** Characteristics (sex, type of sphere, number of lesions, their volumes and strategy of treatment) of patients included in this study

Patient number	Patient sex	Type of spheres	Strategy of treatment	SPECT number	Number of lesion(s) delineated	Volumes (mL)
1	M	Glass	HCC/Lobectomy	1	1	30.89
2	F	Glass	mCRC/Bilobar	2	NA	NA
3	F	Resin	HCC/Lobectomy	3	NA	NA
4	F	Glass	MBC/Lobectomy	4	NA	NA
5	F	Glass	mCRC/Lobectomy	5	NA	NA
6	F	Resin	mCRC/Lobectomy	6	NA	NA
7	M	Glass	HCC/Lobectomy	7	1	54.89
8	M	Glass	MBC/Lobectomy	8	NA	NA
9	F	Glass	MBC/Lobectomy	9	1	176
10	F	Glass	MBC/Lobectomy	10	5	35.54/7.004/11.56/6.331
11	F	Resin	MBC/Lobectomy	11	1	63.74
12	F	Resin	MBC/Lobectomy	12	NA	NA
13	M	Glass	HCC/Lobectomy	13	3	436.6/44.83/432.5 (nodules)
14	M	Glass	MLC/Lobectomy	14	NA	NA
15	M	Glass	ICC/Lobectomy	15	1	228.6
16	M	Resin	HCC/Lobectomy	16	1	278
				17	NA	NA
17	M	Glass	ICC/Lobectomy	18	1	223
				19	NA	NA
18	F	Resin	MLC/Lobectomy	20	1	3.444
19	M	Resin	HCC/Lobectomy	21	1	428.7
20	M	Resin	HCC/Lobectomy	22	2	105.2/23.35
21	M	Glass	HCC/Lobectomy	23	NA	NA
22	F	Glass	mCRC/Lobectomy	24	1	14.15
23	M	Glass	M/Lobectomy	25	1	1828
24	F	Resin	MBC/Lobectomy	26	1	213.9
25	M	Resin	HCC/Lobectomy	27	1	37.01
26	M	Glass	HCC/Lobectomy	28	2	12.99/225.8
27	F	Glass	MPC/Lobectomy	29	4	121.4/106.6/92.65/46.76
28	F	Resin	MNT/Lobectomy	30	1	106.1
29	M	Glass	ICC/Lobectomy	31	2	13.94/19.38

*MBC* metastasis of breast cancer, *MLC* metastasis of lung cancer, *M* metastasis, *MPC* metastasis of pancreatic cancer, *MNT* metastasis of neuroendocrine tumors, *HCC* hepatocellular carcinoma, *mCRC* colorectal cancer metastases, *ICC* intrahepatic cholangiocarcinoma

**Table 2:** DAD calculated for the liver and the lungs for the two reconstructions (3D and 3Dcomp)

	3D reconstruction (Gy)	3Dcomp reconstruction (Gy)
*DAD(Lungs)*		
Minimum	− 0.0032	− 0.00019
1st quartile	5.4e^−6^	8.3e^−6^
Median	0.0036	0.0024
Mean	0.13	0.15
3rd quartile	0.11	0.15
Maximum	1.2	1.1
*DAD(Liver)*		
Minimum	− 0.77	− 2.9
1st quartile	− 0.19	0
Median	0.021	0.0032
Mean	0.16	− 0.034
3rd quartile	0.19	0.12
Maximum	1.8	0.48

For each, the minimum, 1st quartile, median, mean, 3rd quartile and maximum DAD are given

**Table 3:** Activities to be prescribed for each patient and each reconstruction (3D and 3Dcomp) estimated from international recommendations [10, 34]

Number of patient	Activity in GBq (3D)	Activity in GBq (3Dcomp)	Recommended dose tolerance limit reached	Relative difference between 3D and 3Dcomp (%)
1	4.263	4.298	Normal liver dose	0.8
7	2.470	2.538	Normal liver dose	2.8
9	13.651	13.051	Normal liver dose	− 4.4
10	1.632	1.496	Normal liver dose	− 8.3
11	1.312	1.362	Tumor dose	3.8
13	6.604	6.582	Normal liver dose	− 0.3
15	3.525	3.553	Normal liver dose	0.8
16	2.907	2.907	Normal perfused liver dose	0
17	4.510	4.495	Normal liver dose	− 0.3
18	1.280	1.572	Tumor dose	22.8
19	2.514	2.530	Normal perfused liver dose	0.6
20	2.324	2.450	Tumor dose	5.4
22	1.202	1.219	Normal liver dose	1.4
23	5.077	5.076	Normal liver dose	0.0
24	5.793	5.907	Normal liver dose	2.0
25	1.227	1.194	Normal perfused liver dose	− 2.7
26	4.694	4.560	Normal liver dose	− 2.9
27	4.251	4.262	Normal liver dose	0.3
28	3.459	3.419	Normal liver dose	− 1.2
29	2.354	2.372	Normal liver dose	0.8

The relative difference of dose was also computed. The criterion corresponded to the limit that led to the choice of this activity to prescribe

**Table 4:** Table of estimated absorbed doses to lungs and tumors from 3D and 3Dcomp reconstructions

Number of patients	Lungs dose Gy (3D)	Lungs dose Gy (3Dcomp)	PDD of lungs (%)	Tumor dose Gy (3D)	Tumor dose Gy (3Dcomp)	PDD of Tumor (%)
1	9.2	9.4	1.9	179.3	180.4	0.6
				245.0 NA	242.4 NA	− 1.1 NA
2	4.4	4.1	− 6.4			
3	1.3	1.1	− 17.2	NA	NA	NA
4	1.3	1.4	2.6	NA	NA	NA
5	1.5	1.7	17.4	NA	NA	NA
6	1.1	1.2	8.4	NA	NA	NA
7	2.2	2.5	10.3	331.4	354.0	6.8
8	2.5	3.0	21.2	NA	NA	NA
9	0.3	0.3	4.0	186.2	184.4	− 1.0
10	8.3	4.3	− 48.6	229.3	284.1	23.9
				147.8	166.1	12.4
				91.5	71.5	− 21.8
				175.6	155.0	− 11.7
				118.7	84.2	− 29.0
11	0.7	0.7	9.7	76.0	73.1	− 3.7
12	4.8	2.1	− 56.9	NA	NA	NA
13				49.3	49.9	1.2
	3.6	3.7	2.9	103.2	109.5	6.1
				105.1	104.8	− 0.3
14	0.6	0.8	28.2	NA	NA	NA
15	3.0	4.2	39.4	170.6	164.5	− 3.6
16	1.4	1.7	20.1	148.4	148.6	0.2
16	0.4	0.5	17.2	NA	NA	NA
17	4.1	3.4	− 17.7	49.0	48.1	− 1.9
17	3.2	3.4	6.0	NA	NA	NA
18	4.4	3.5	− 20.3	88.4	72.0	− 18.6
19	8.1	8.8	8.8	190.3	186.7	− 1.8
20	1.3	1.6	29.8	73.2	69.4	− 5.1
				116.1	116.1	− 0.02
21	0.2	0.2	6.8	NA	NA	NA
22	2.4	2.7	11.0	182.0	183.2	0.7
23	8.3	8.9	6.4	41.3	40.8	− 1.2
24	3.9	4.1	3.7	173.3	175.3	1.2
25	1.8	1.1	− 41.1	157.4	158.9	0.9
26	6.5	3.7	− 43.3	297.5	354.8	19.2
				258.6 12.5	258.4 11.1	− 0.1 − 11.3
27	2.7	3.0	10.0	114.1	111.0	− 2.7
				49.2 166.1	49.9 164.1	1.5 − 1.2
28	2.5	2.0	− 20.4	76.4	73.9	− 3.3
29	1.9	2.4	26.2	152.7	154.4	1.1
				116.4	116.4	− 0.01

For each patient and volume, the PDDs were also calculated

*NA* tumor contours are not available
